# Redefining Myeloid Cell Subsets in Murine Spleen

**DOI:** 10.3389/fimmu.2015.00652

**Published:** 2016-01-11

**Authors:** Ying-Ying Hey, Jonathan K. H. Tan, Helen C. O’Neill

**Affiliations:** ^1^Research School of Biology, Australian National University, Canberra, ACT, Australia; ^2^Faculty of Health Sciences and Medicine, Bond University, Robina, QLD, Australia

**Keywords:** dendritic cells, monocytes, spleen, myeloid cells

## Abstract

Spleen is known to contain multiple dendritic and myeloid cell subsets, distinguishable on the basis of phenotype, function and anatomical location. As a result of recent intensive flow cytometric analyses, splenic dendritic cell (DC) subsets are now better characterized than other myeloid subsets. In order to identify and fully characterize a novel splenic subset termed “L-DC” in relation to other myeloid cells, it was necessary to investigate myeloid subsets in more detail. In terms of cell surface phenotype, L-DC were initially characterized as a CD11b^hi^CD11c^lo^MHCII^−^Ly6C^−^Ly6G^−^ subset in murine spleen. Their expression of CD43, lack of MHCII, and a low level of CD11c was shown to best differentiate L-DC by phenotype from conventional DC subsets. A complete analysis of all subsets in spleen led to the classification of CD11b^hi^CD11c^lo^MHCII^−^Ly6C^lo^Ly6G^−^ cells as monocytes expressing CX_3_CR1, CD43 and CD115. Siglec-F expression was used to identify a specific eosinophil population, distinguishable from both Ly6C^lo^ and Ly6C^hi^ monocytes, and other DC subsets. L-DC were characterized as a clear subset of CD11b^hi^CD11c^lo^MHCII^−^Ly6C^−^Ly6G^−^ cells, which are CD43^+^, Siglec-F^−^ and CD115^−^. Changes in the prevalence of L-DC compared to other subsets in spleens of mutant mice confirmed the phenotypic distinction between L-DC, cDC and monocyte subsets. L-DC development *in vivo* was shown to occur independently of the BATF3 transcription factor that regulates cDC development, and also independently of the FLT3L and GM-CSF growth factors which drive cDC and monocyte development, so distinguishing L-DC from these commonly defined cell types.

## Introduction

Characterization of dendritic cell (DC) subsets in spleen has progressed rapidly in terms of phenotype and function; however, other splenic myeloid subsets are less clearly defined. The term “myeloid” has been used as an umbrella term describing DC, granulocytes, and macrophages/monocytes, which descend from a common myeloid progenitor (CMP) in bone marrow (1). Granulocytes comprise neutrophils, eosinophils, basophils, and mast cells (2), while monocytes have been classified into distinct resident and inflammatory populations, in line with monocyte subsets described in peripheral blood (3). Recent definition of a common dendritic progenitor (CDP) now separates the development of conventional (c) and plasmacytoid (p) DC from other myeloid cell types (4, 5). The current classification of splenic myeloid subsets is based on cell surface phenotype, although full phenotypic profiles are not yet available. To date, studies focusing on specific subsets do not compare one subset against another to ensure pure populations and do not achieve a comprehensive picture of the relationship between subsets. Accurate identification of splenic myeloid cell types is essential for distinguishing specific subsets and their function, and for making studies in the field comparable.

Monocytes develop in bone marrow from CMP and traverse into blood as mature cells (1). Resident monocytes were described as having a longer half-life in blood than inflammatory monocytes (6) and were originally thought to act as precursors of tissue resident macrophages in the steady-state, hence their name “resident” monocytes (7). They were identified further as CC-chemokine receptor 2 (CCR2)^−^ and CX3-chemokine receptor 1 (CX_3_CR1)^hi^Ly6C^lo^Ly6G^−^ cells, which play an important role in the detection of vascular integrity (3, 8). Inflammatory monocytes were described as distinct as CCR2^+^CX_3_CR1^lo^Ly6C^hi^Ly6G^−^ cells (3, 7, 9–11). Several studies have now highlighted how tissue-specific macrophages including microglial cells, alveolar macrophages, Langerhans cells and splenic macrophages have a yolk sac-derived origin rather than a bone marrow origin (12–15).

Most studies on monocytes have involved blood as a source of cells, but more recently the relationship between splenic monocytes and their blood counterpart has been considered. The studies of Swirski and colleagues demonstrated that splenic monocytes resemble their blood counterpart on the basis of phenotype, morphology and gene expression (16). Spleen was identified as a reservoir for monocytes, where Ly6C^hi^ inflammatory monocytes were efficiently deployed to sites of inflammation during cardiac arrest. Monocytes in blood and spleen are now identified as cells with a CD11b^hi^CD11c^−/lo^ phenotype, with further distinction as Ly6C^hi^ and Ly6C^lo^ cells resembling inflammatory and residential monocytes, respectively (17). Neutrophils have not been extensively characterized in spleen and were previously delineated on the basis of phenotype as a blood equivalent CD11b^+^Ly6C^+^Ly6G^+^ subset (18). Similarly, eosinophils have been described in blood as CD11b^+^Siglec-F^+^CCR3^+^F4/80^+^ cells and in spleen as Ly6C^int^Ly6G^−^SSC^hi^Siglec-F^+^ cells (17, 19).

The lineage of DC is equally diverse with multiple subsets defined in mice and humans (20, 21). All subsets are capable of antigen uptake, processing and presentation for T cell activation. Conventional DC are predominant in spleen, comprising CD8^−^ and CD8^+^ populations, phenotypically distinct as CD11c^hi^CD11b^+^CD8α^−^MHCII^+^ and CD11c^hi^CD11b^−^CD8α^+^MHCII^+^ cells, respectively (22). These two dominant DC subsets are distinct in function, cytokine production and ability to cross-present antigen (23). Spleen also contains a smaller population of pDC existing as a plasmacytoid preDC (p-preDC) in the steady-state. These are, however, long-lived, circulating cells, which produce high levels of Type I interferon important in anti-viral immunity (24). Spleen also contains a variable population of poorly defined “myeloid DC.” As an example, under inflammatory conditions, circulating CD115^+^Ly6C^hi^CCR2^+^ monocytes in blood may be recruited into spleen where they differentiate to become “monocyte-derived (mo)-DC” (25–27). Different subsets of mo-DC can subsequently be observed in different disease states.

Novel dendritic-like cells termed L-DC, were recently discovered in murine and human spleen on the basis of their resemblance with dendritic-like cells produced *in vitro* in splenic long-term cultures (LTC-DC) (28–30), or in co-cultures of bone marrow precursors over selected splenic stroma (31, 32). L-DC have a distinct CD11b^hi^CD11c^lo^MHCII^−^CD8^−^ phenotype and dendritic-like appearance (33). Splenic stroma maintains continuous but restricted *in vitro* development of only this cell type, without addition of cytokines like GM-CSF, M-CSF, or Flt3L used by others to induce DC development *in vitro* from bone marrow precursors (34). An *in vivo* equivalent L-DC subset has been partially characterized in spleens of mice and human (35, 36). While L-DC resemble myeloid DC on the basis of CD11c and CD11b expression, lack of MHCII expression has raised criticism that L-DC may more resemble splenic monocytes/macrophages than DC. Low expression of CD11c on L-DC is, however, consistent with some myeloid DC and pDC described as CD11c^lo^ cells (37–39).

Analysis of cell surface phenotype using specific antibodies and flow cytometry is widely accepted as a means to identify and distinguish *ex vivo* cell subsets. A staining protocol and gating strategy were therefore developed here to more accurately delineate and identify DC, monocytes, and other myeloid subsets in spleen. This study now identifies the L-DC subset *in vivo* as distinct from monocytes, granulocytes and cDC. The development of L-DC has also been investigated in relation to cDC and myeloid subsets in the spleens of *Batf-3*^−/−^, *GM-CSF*^−/−^, and *Flt3L*^−/−^ mutant mice. The GM-CSF and FLT3 ligand (FLT3L) growth factors are known to be important in the differentiation of dendritic and other myeloid cells (34), and the BATF3 transcription factor was recently described as essential for CD8^+^ cDC development (40, 41).

## Materials and Methods

### Animals

C57BL/6J mice were bred at the John Curtin School of Medical Research (JCSMR: Canberra, ACT, Australia) under specific pathogen-free conditions. B6.129P(Cg)-*Ptprc^*a*^*Cx3Cr1*^*tm1Litt*^*LittJ(*Cx3Cr1-GFP*) mice were purchased from the Walter and Eliza Hall Institute (Parkville, VIC, Australia). C57BL/6-*Flt3L*^tm1lmx^ (*Flt3L*^−^*^*/*^*^−^) mice (Taconic Farms Inc., NY, USA) were purchased from the Biomedical Research Facility, University of Western Australia (Perth, WA, Australia). C57BL/6-*Csf2^*tm1Ard*^* (*GM-CSF*^−^*^*/*^*^−^) mice were obtained from the Ludwig Institute for Cancer Research (Melbourne, VIC, Australia). C57BL/6-129S-*Batf^*tm1.1Kmm*^* (*Batf-3*^−^*^*/*^*^−^) mice were provided through the courtesy of Ian Cockburn (JCSMR). Mice were housed and handled according to the guidelines of the Animal Experimentation Ethics Committee at the Australian National University (Canberra, ACT, Australia).

### Cell Fractionation

Dendritic and myeloid cells were enriched from dissociated whole spleen via negative depletion of T, B, and red blood cells using magnetic bead separation and MACS^®^ technology (Miltenyi Biotec: Auburn, CA, USA). Depletion was performed by exposing cells to specific antibody, i.e., 0.2 μg biotinylated anti-Thy1.2, anti-CD19, and anti-Ter119 antibody/10^8^ cells in 1 mL of MACS-labeling buffer (2 mM EDTA/0.5% BSA in PBS) on ice for 25 min. Cells were then washed twice with MACS-labeling buffer, resuspended in buffer (10^8^ cells/mL) followed by the addition of 20 μL of anti-biotin microbeads/10^8^ cells (Miltenyi), with incubation for 25 min on ice. The washing step was repeated, and cells resuspended in MACS-labeling buffer. T, B, and red blood cells were depleted by running cells through LS columns (Miltenyi) in a SuperMACS II Separation Unit (Miltenyi), washing thrice with 3 mL of buffer and collection of unbound cells as flow-through.

### Antibody Staining

**Figure 1:**
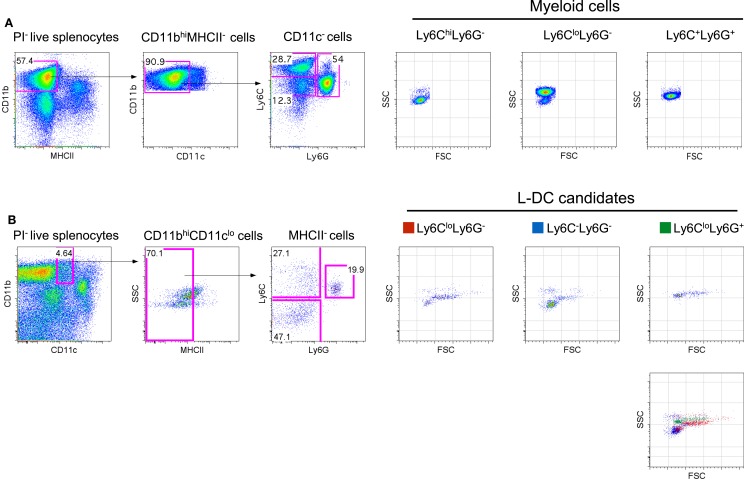
**Phenotypic characterization of myeloid subsets in spleen**. Spleen cells were depleted of T, B and red blood cells and stained with antibodies to CD11b, CD11c, MHCII, Ly6C, and Ly6G. Propidium iodide (PI, 1 μg/mL) staining was used to gate live (PI^−^) cells. **(A)** CD11b^hi^CD11c^−^MHCII^−^ cells were initially gated on the basis of a blood monocyte phenotype. Further staining with Ly6C and Ly6G revealed three subpopulations with distinct FSC and SSC as reported in the literature (42). **(B)** L-DC were initially gated as CD11b^hi^CD11c^lo^MHCII^−^ cells. Further staining with Ly6C and Ly6G revealed three populations of “L-DC candidates” with overlapping FSC and SSC profiles: Ly6C^lo^Ly6G^+^, Ly6C^lo^Ly6G^−^ and Ly6C^−^Ly6G^−^. Gating strategies were based on fluorescence minus one control, and numbers in gates represent % specific binding.

**Figure 2:**
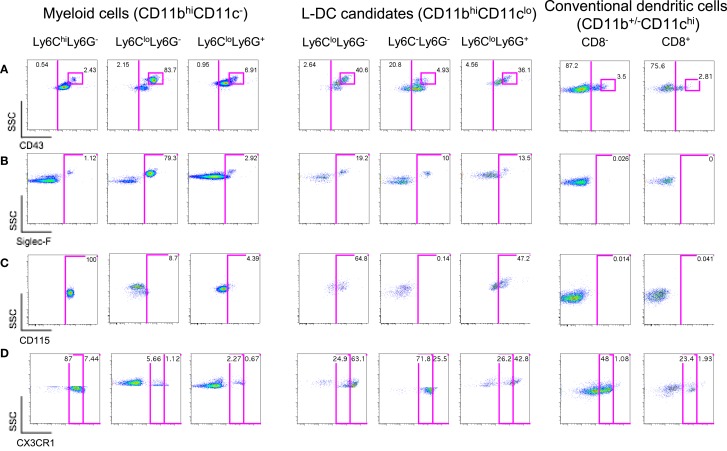
**Further delineation of myeloid cells on the basis of CD43, Siglec-F, and CD115 expression**. Spleen cells were prepared and stained as described in Figure 1 with the addition of antibodies specific for CD43, CD115, Siglec-F, and CX3CR1. Live (PI^−^) cells were gated as myeloid cells (CD11b^hi^CD11c^−^) and L-DC candidates (CD11b^hi^CD11c^lo^). Myeloid cells were further divided to reveal Ly6C^hi^Ly6G^−^, Ly6C^lo^Ly6G^−^, and Ly6C^lo^Ly6G^+^ subsets. L-DC were further delineated as three candidate subsets differing in Ly6C and Ly6G expression. Expression of **(A)** CD43, **(B)** Siglec-F, **(C)** CD115, and **(D)** CX3CR1 was determined on all subsets. Gates were set based on fluorescence minus one control, and numbers in gates represent % specific binding.

**Figure 3:**
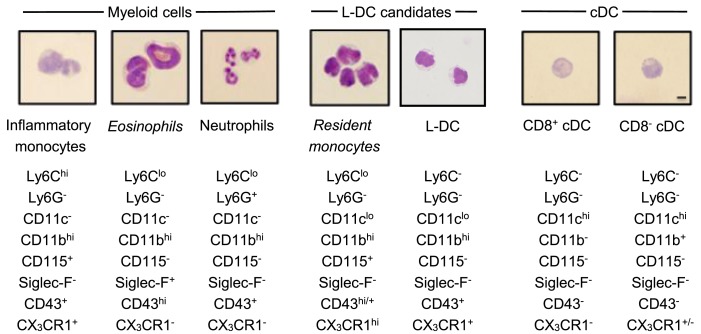
**Morphology of spleen dendritic and myeloid subsets**. Splenocytes were sorted into myeloid subsets, L-DC candidates and cDC as described in Figure 2. Sorted subsets were cytospun on to slides and fixed prior to Giemsa staining. Photomicroscopy of subsets was performed at 600× magnification, bar 10 μm. Italicized labels indicate revised subset classification based on identified morphology and cell surface phenotype. Ly6C^hi^ and Ly6C^lo^ monocytes are labeled as inflammatory and resident monocytes, respectively.

**Figure 4:**
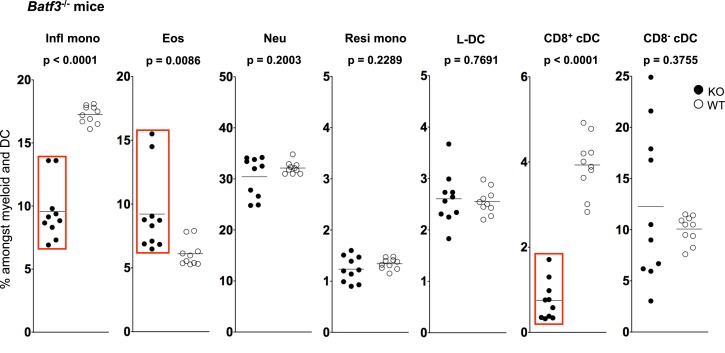
**L-DC development occurs independently of Batf-3 signaling**. Splenocytes were harvested from B6.129S(C)-*Batf3^tm1.1Kmm^* (Batf-3^−/−^) and C57BL/6J (wild-type) mice and prepared as described in Figure 1. Cells were stained with two distinct antibody cocktails. The first included antibodies to CD11b, CD11c, Ly6C, Ly6G, MHC-II and CD8. The second contained antibodies to CD11b, CD11c, Ly6C, Ly6G, CD43 and Siglec-F. Prior to analysis, cells were stained with propidium iodide for gating live (PI^−^) cells. CD11b^hi^CD11c^−^ myeloid cells were gated to give Ly6C^hi^Ly6G^−^CD43^+^Siglec-F^−^-inflammatory monocytes (Infl mono), Ly6C^lo^Ly6G^−^CD43^+^Siglec-F^+^ eosinophils (Eos) and Ly6C^lo^Ly6G^+^CD43^+^Siglec-F^−^ neutrophils (Neu). L-DC were gated as CD11b^hi^CD11c^lo^Ly6C^−^Ly6G^−^CD43^+^Siglec-F^−^ cells, while CD11b^hi^CD11c^lo^Ly6C^lo^Ly6G^−^CD43^hi^Siglec-F^−^ cells were gated as resident monocytes (Resi mono). CD8^+^cDC were gated as CD11b^−^CD11c^hi^MHCII^+^CD8^+^Ly6C^−^Ly6G^−^ cells, while CD8^−^cDC were gated as CD11b^+^CD11c^hi^MHCII^+^CD8^−^Ly6C^−^Ly6G^−^ cells. Subset size was estimated as % of the total DC and myeloid population in individual wild-type mice (WT) compared with *Batf3*^−^*^/^*^−^ mice (KO). A bar is used to show mean values and *p* values are given. Statistically different subsets (*p* ≤ 0.05) are boxed.

Antibody staining followed by flow cytometry was performed to analyze cell surface marker expression as described previously (32). Non-specific antibody binding via Fc receptors was blocked by incubating cells (≤ 10^6^) with anti-CD16/32 (FcBlock) (Biolegend: San Diego, CA, USA) at 5 μg/mL. Biotin- or fluorochrome-conjugated antibodies specific for CD11c (N418), CD11b (M1/70), CD8 (53-6.7), CD19 (1D3), CD43 (IBII), Thy1.2 (30-H12), Ter119 (Ter-119), F4/80 (CI:A3-1), IA^b^ (25-9-17), Siglec-F (E50-2440), Ly6C (HK1.4), and Ly6G (1A8) were purchased from Biolegend. Antibody specific for CD115 (AFS98) was purchased from eBioscience (Parkville, VIC, Australia). Propidium iodide (PI, 1 μg/mL) was added prior to flow cytometry for discrimination of live and dead cells. Flow cytometry was performed immediately on a BD LSRII flow cytometer (Becton Dickinson: Franklin Lakes, NJ, USA). Data collected included forward scatter (FSC), side scatter (SSC), and multiple fluorescence channels detecting fluorescein isothiocyanate (FITC), phycoerythrin (PE), PI, pacific blue (PB), Alexa Fluor-700, PE-indocyanine-7 (Cy7), allo-phycocyanin (APC), and APC-Cy7. BD FACSDiva Software (Becton Dickinson) was used to acquire data. Analysis of data involved post-acquisition gating using FlowJo software (Tree Star: Ashland, OR, USA). Analyses shown in Figures 1–4 were repeated at least thrice, but only representative data are shown. Replicates are shown for all other experiments.

### Cell Sorting

Cell populations were isolated by sorting following flow cytometry with fluorochrome-conjugated antibodies. Cells were prepared as described above and all incubation and washing steps performed in 1% fetal calf serum in Dulbecco’s Modified Eagle Medium (DMEM). After a final wash prior to sorting, cells were filtered through a 70-μm nylon cell strainer (Becton Dickinson) for removal of cell clumps. Cell sorting was performed using a FACSAria cell sorter (Becton Dickinson). Sorted populations were collected in complete medium (10% fetal calf serum in DMEM) as described previously (32).

### May-Grünwald-Giemsa Staining

Cell staining with Giemsa was employed to morphologically differentiate cells within sorted populations. Sorted cells (10^3^–10^6^) were pelleted on to a glass slide using a cytospin centrifuge. Cells were fixed in methanol, then stained in a two-step procedure with CliniPure staining solution 1 (0.25% Eosin YO/Soresen buffer, pH 6.8) followed by staining solution 2 (0.25% methylene blue polychrone/Soresen buffer, pH 6.8) (HD Scientific: Sydney, NSW, Australia) for 5 s at each step. Excess solution was rinsed off and slides dried before mounting. Non-aqueous Depex mounting agent (Fluka Analytical: Buchs, Switzerland) was used to prevent leaching of dye from stained cells. Photographs were taken with a LEICA DFC digital camera connected to a LEICA brightfield inverted microscope (LEICA Microsystems: Wetzlar, Germany).

### Statistical Analysis

Data have been presented as mean ± SE for sample size *n*. For sample size *n* ≤ 5, the Wilcoxon Rank-Sum test was used to test significance (*p* ≤ 0.05). Where a normal distribution could be assumed (*n* > 5), Students’ *t*-test was used to determine significance (*p* ≤ 0.05).

## Results

### Identification of Myeloid Subsets in Spleen

In blood, both the described populations of Ly6C^hi^ inflammatory and Ly6C^lo^-resident monocytes do not express Ly6G (3). Ly6C^hi^ monocytes are distinct from neutrophils which express Ly6G as well as Ly6C. In order to identify myeloid cells in spleen, CD11b^hi^CD11c^−^MHCII^−^ cells were initially gated, then further delineated by Ly6C and Ly6G expression to yield subsets with a Ly6C^hi^Ly6G^−^, Ly6C^lo^Ly6G^−^, and Ly6C^+^Ly6G^−^ phenotype as previously described in blood (Figure 1A). These myeloid subsets were then distinguishable on the basis of SSC, with the Ly6C^hi^ subset as SSC^lo^ cells, Ly6C^lo^ cells as SSC^hi^ and neutrophils as SSC^mid^ (Figure 1A). Staining with antibodies specific for CD11b, CD11c, MHCII, Ly6C and Ly6G, and analysis of SSC can therefore be used to distinguish three main myeloid subsets in spleen, equivalent to those described previously in blood (7, 25).

The novel L-DC subset resembles myeloid DC in spleen on the basis of high CD11b expression. In order to further explore the relationship between L-DC and the three major myeloid subsets described above, T, B, and red blood cell-depleted splenocytes were gated as CD11b^hi^CD11c^lo^MHCII^−^ cells, and further tested for Ly6C and Ly6G expression. Since three distinct populations of cells were observed, Ly6C^+^Ly6G^+^ (19.9%), Ly6C^+^Ly6G^−^ (27.1%), and Ly6C^−^Ly6G^−^ (47.1%) (Figure 1B), these were selected for further study and termed “L-DC candidates.” FSC and SSC profiles distinguished Ly6C^+^Ly6G^+^ and Ly6C^+^Ly6G^−^ candidates as FSC^hi^SSC^mid^, while the Ly6C^−^Ly6G^−^ subset was predominantly FSC^lo^SSC^lo^ (Figure 1B). To fully delineate the L-DC subset and to distinguish it from myeloid cells, it was necessary to identify further markers and to characterize all myeloid subsets concurrently.

Ly6C^lo^-resident monocytes in blood were previously distinguished from Ly6C^hi^-inflammatory monocytes on the basis of higher CD43 expression (10). In addition, eosinophils present in steady-state spleen were distinguishable from other myeloid cells through expression of Siglec-F (17, 19). Myeloid cells identified in Figure 1 were therefore stained for these markers. All CD11b^hi^CD11c^−^ cells delineated by Ly6C and Ly6G expression expressed CD43 (Figure 2A). The majority of Ly6C^hi^Ly6G^−^ monocytes (~99%) and Ly6C^+^Ly6G^+^ neutrophils (~97%) did not express Siglec-F (Figure 2B). The Ly6C^lo^Ly6G^−^ population contained a distinct CD43^hi^ subset (83.7%), which corresponded to SSC^hi^Siglec-F^+^ cells and therefore resembled eosinophils (Figures 2A,B). All L-DC candidates, irrespective of Ly6C/Ly6G delineation, expressed CD43 (Figure 2A). The Ly6C^lo^Ly6G^−^ candidates displayed the largest CD43^hi^ population (40.6%), also a reflection of resident monocytes. While the majority of cells in all subsets were Siglec-F^−^, each Ly6C^−^Ly6G^−^, Ly6C^+^Ly6G^−^, and Ly6C^+^Ly6G^+^ subset showed some low level contamination with Siglec-F^+^ eosinophils (Figure 2B).

The presence of Siglec-F^+^ cells among splenic myeloid cells therefore necessitated mapping out the eosinophil phenotype in relation to myeloid cells. As shown in Figure 2B, Ly6C^lo^Ly6G^−^ myeloid cells contained a large subset of Siglec-F^+^ cells (79.3%), indicating a majority of eosinophils. The presence of eosinophils was also confirmed by transcriptome analysis, which revealed specific expression of CCR3 by this subset (unpublished data). In order to confirm the specificity of Siglec-F staining, a back-gating strategy was applied to Siglec-F^+^ cells (Figure S1 in Supplementary Material). Siglec-F^+^ eosinophils were found to be mainly CD11b^hi^CD11c^−^Ly6C^lo^Ly6G^−^CD43^+^ cells. Since this phenotype also reflects Ly6C^lo^ monocytes in blood, future stainings therefore involved Siglec-F gating to distinguish or exclude eosinophils from splenic subsets of interest.

The expression of macrophage-colony stimulating factor receptor (M-CSFR/CD115) has also proven useful to delineate monocytes/macrophages and other myeloid subsets (43, 44). Here, it was found that Ly6C^lo^Ly6G^−^Siglec-F^+^ eosinophils were CD115^−^, while Ly6C^hi^Ly6G^−^ monocytes were CD115^+^ (Figure 2C). Ly6C^+^Ly6G^+^ neutrophils were also CD115^−^, consistent with reports in the literature (45). Among L-DC candidates, 65% of the Ly6C^lo^Ly6G^−^ subset, now thought to contain monocytes, expressed CD115. The Ly6C^+^Ly6G^+^ subset of L-DC candidates, thought to be contaminated with neutrophils and other undefined cells, showed 47% of cells as CD115^+^. However, the Ly6C^−^Ly6G^−^ subset of L-DC candidates was clearly negative for CD115, distinguishing it from other CD11b^hi^CD11c^lo^ L-DC candidates (Figure 2C).

### Redefining Splenic Myeloid Subsets

Myeloid subsets in spleen were investigated further in terms of the expression of specific markers which are known to distinguish myeloid cells. CX_3_CR1 expression has been associated with the differentiation of macrophages and DC from a macrophage/dendritic progenitor (MDP) (46–48), and Ly6C^lo^-resident monocytes and Ly6C^hi^-inflammatory monocytes in blood have been described as CX_3_CR1^hi^ and CX_3_CR1^lo^ cells, respectively (3, 7). CX_3_CR1 transgenic mice tagged with green fluorescence protein (GFP) were therefore used to confirm that the subset of CD11b^hi^CD11c^−^Ly6C^lo^Ly6G^−^Siglec-F^+^ cells did not express CX_3_CR1 in line with the designation of eosinophils. When splenic SSC^hi^Ly6G^−^ cells corresponding to CD11b^hi^CD11c^−^Ly6C^lo^Ly6G^−^Siglec-F^+^ cells were gated, very few cells were found to express CX_3_CR1 (6.78%; Figure 2D), consistent with their classification as eosinophils rather than monocytes. The gated CD11b^hi^CD11c^−^Ly6C^hi^Ly6G^−^ cells were found to be CX_3_CR1^lo^, so confirming their identity as Ly6C^hi^-inflammatory monocytes described by Geissmann et al. (7) (Figure 2D). Finally, Ly6G^+^Ly6C^+^ cells were found to be CX_3_CR1^−^ and so were likely to be neutrophils.

L-DC candidates were then analyzed for CX_3_CR1 expression. For this experiment, they were divided into three populations based on SSC and Ly6G staining, and Ly6C expression on candidate subsets gated on the basis of SSC and Ly6G was also verified by independent staining (data not shown). Of the three L-DC candidate subsets, SSC^hi^Ly6C^lo^Ly6G^−^ cells were largely CX_3_CR1^hi^ (63%), and along with their CD115 expression, reflected a phenotype consistent with Ly6C^lo^ monocytes (Figure 2D). The Ly6C^+^Ly6G^+^ subset classified as neutrophils appeared heterogeneous with 43% CX_3_CR1^hi^ cells. The SSC^lo^Ly6C^−^Ly6G^−^ L-DC candidate, negative for CD115 expression, was also CX_3_CR1^+^. Low CX_3_CR1 expression was also observed on 48% of gated CD8^−^ cDC and 23% of gated CD8^+^ cDC (Figure 2D), consistent with previous reports (49, 50). In terms of L-DC, the expression of CX_3_CR1 on SSC^lo^Ly6C^−^Ly6G^−^ cells is consistent with a dendritic/monocyte cell type.

### L-DC Are Morphologically Distinct from Monocytes

In order to classify subsets further, myeloid and DC subsets were sorted to purity, prepared as cytospins, and visualized by Giemsa staining. Light microscopy was used to identify and quantitate cells based on morphology. Among CD11b^hi^CD11c^−^ myeloid cells, the Ly6C^+^Ly6G^+^ subset (also 7/4^+^Siglec-F^−^) demonstrated characteristic neutrophil morphology, with a multi-lobate nucleus and cytoplasm devoid of granules (Figure 3). Over two independent experiments, all cells counted from this subset showed similar neutrophil morphology (Table 1). Giemsa staining of sorted CD11b^hi^CD11c^−^Ly6C^hi^Ly6G^−^ (SiglecF^−^7/4^−^) cells revealed a bi-lobate nucleus and a cytoplasm devoid of granules (Figure 3). The majority of cells (>90%) displayed this morphology (Table 1), confirming the phenotype of Ly6C^hi^-inflammatory monocytes. Gated CD11b^hi^CD11c^−^Ly6C^lo^Ly6G^−^ (SiglecF^+^7/4^−^) cells demonstrated multi-lobate nuclei with clear presence of orange granules in the cytoplasm, consistent with eosinophils and confirming a Siglec-F^+^ identity (Figure 2).

**Table 1:** **Morphological characterization of spleen dendritic and myeloid subsets**.

Sorted subset	Subset classification	Expt^a^	No. of cells	% cells with known morphology^b^			
				Eosinophil	Neutrophil	Monocyte-like	Dendritic-like
CD11b^hi^CD11c^−^Ly6C^hi^Ly6G^−^CD43^+^Siglec-F^−^	Inflammatory monocytes	I	72	1.4	–	**97.2^c^**	1.4
		II	68	1.5	–	**86.8**	11.8
CD11b^hi^CD11c^−^Ly6C^lo^Ly6G^−^CD43^hi^Siglec-F^+^	Eosinophils	I	72	**100**	–	–	–
		II	72	**100**	–	–	–
CD11b^hi^CD11c^−^Ly6C^lo^Ly6G^+^CD43^+^Siglec-F^−^	Neutrophils	I	26	–	**100**	–	–
		II	18	–	**100**	–	–
CD11b^hi^CD11c^lo^Ly6C^lo^Ly6G^−^CD43^+/hi^Siglec-F^−^	Resident monocytes	I	76	–	–	**68.4**	31.6
		II	80	–	–	**62.5**	37.5
CD11b^hi^CD11c^lo^Ly6C^−^Ly6G^−^CD43^+^Siglec-F^−^	L-DC	I	78	10.3	–	42.3	**47.4**
		II	84	6.0	–	32.1	**62**
CD11b^−^CD11c^hi^Ly6C^−^Ly6G^−^CD43^−^Siglec-F^−^	CD8^+^ cDC	I	41	–	–	26.8	**73.2**
		II	34	–	–	17.7	**82.4**
CD11b^+^CD11c^hi^Ly6C^−^Ly6G^−^CD43^−^Siglec-F^−^	CD8^−^ cDC	I	61	–	–	13.1	**86.9**
		II	59	1.7	–	15.3	**83.1**

*^a^Data are shown for two independent sorting experiments*.

*^b^Cells were cytospun and stained with Giemsa for microscopic classification*.

***^c^**Dominant subsets are shown in bold*.

Among the CD8^+^ and CD8^−^ cDC subsets, a majority (80–90%) of cells displayed the morphology of mononuclear dendritic-like cells with vacuoles evident in the cytoplasm and nuclei staining blue (Table 1; Figure 3). Among the CD11b^hi^CD11c^lo^ candidates, the Ly6C^+^Ly6G^−^ subset revealed morphology resembling monocytes with a bi-lobate nucleus and a cytoplasm devoid of granules (Figure 3). Combined with a SiglecF^−^CD43^+/hi^CX_3_CR1^hi^CD115^+^ phenotype, this population is reflective of Ly6C^lo^ monocytes. A subpopulation of ~30% cells, however, was observed to show morphology more typical of DC (Table 1), and this could indicate an impure population. The Ly6C^+^Ly6G^+^ subset comprised mainly neutrophils, with ~10% monocytes (data not shown). Sorted Ly6C^−^Ly6G^−^ L-DC were also heterogeneous, with the presence of both dendritic-like and monocyte-like cells. Across two experiments, 47 and 62% of cells displayed a mononuclear dendritic-like morphology with veiled membranes, short dendrites, and vacuoles evident in the cytoplasm, with 30–40% of cells showing more monocyte-like morphology (Table 1; Figure 3). Five to ten percent of cells represented eosinophils as a potential contaminant identified in Figure 2. Overall, Giemsa staining revealed the presence of a majority of dendritic-like cells in the CD11b^hi^CD11c^lo^Ly6C^−^Ly6G^−^ subset, and based on collective data, this subset most likely represents L-DC, with monocytes representing the major subpopulation of Ly6C^+^Ly6G^−^ cells, and neutrophils comprising most of the Ly6C^+^Ly6G^+^ subset.

### L-DC Are a Distinct Lineage from cDC

While L-DC are phenotypically distinct from cDC, they show similarity in their endocytic and cross-presentation ability (36). To determine if L-DC and cDC derive from a common lineage progenitor, their prevalence was investigated in *Batf3*^−/−^ mice. The expression *Batf3* has been described for pre-cDC, with peak expression in differentiated CD8^+^ and CD8^−^ cDC (51–53). *Batf3*^−/−^ mice contain decreased numbers of CD8^+^ cDC, but not CD8^−^ cDC, suggesting that *Batf3* is essential in the development of CD8^+^ cDC from pre-cDC (51). *Batf3* may co-operate with another factor to induce the final differentiation of CD8^+^ cDC, while CD8^−^ cDC differentiation may occur independently of *Batf3* (52).

**Figure 5:**
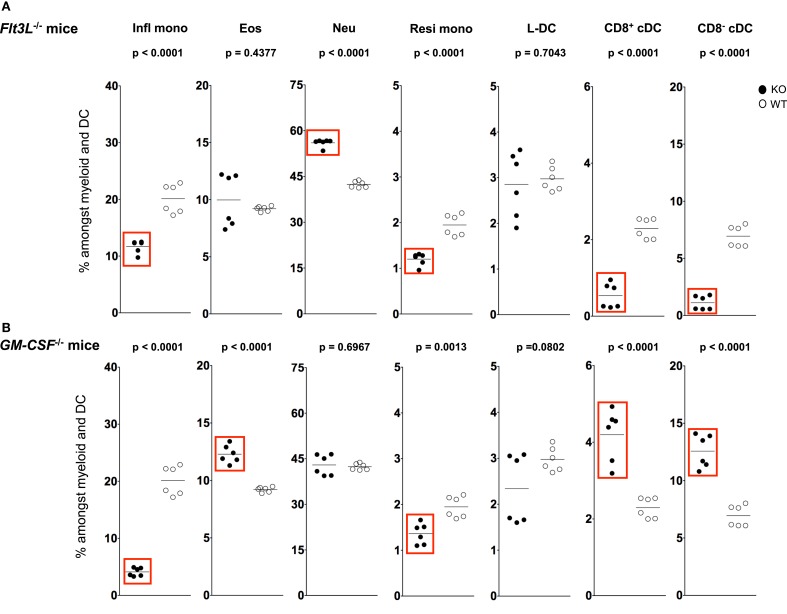
**L-DC development occurs independently of Flt3L and GM-CSF**. Splenocytes were harvested from C57BL/6J-*Flt3L^tm1lmx^* (Flt3L^−/−^), C57BL/6J-*Csf2^tm1Ard^* (GM-CSF^−/−^), and C57BL/6J (wild-type) mice, and prepared as described in Figure 1. Cells were stained with two distinct antibody cocktails and propidium iodide (PI) as described in Figure 4. Subset size was estimated as % of total DC and myeloid population in individual wild-type mice (WT) compared with **(A)** *Flt3L*^−^*^/^*^−^ and **(B)** *GM-CSF*^−^*^/^*^−^ mice (KO). A bar is used to show mean values and *p* values are given. Statistically different subsets (*p* ≤ 0.05) are boxed.

The proportional representation of dendritic and other myeloid subsets in spleen relative to total splenic myeloid subsets of CD11b^+^ and/or CD11c^+^ cells was compared in mutant and wild-type mice. Subsets were delineated as described in Figure 3 and Table 1. A significant drop in the number of CD8^+^ cDC was observed in *Batf3*^−/−^ mice compared with wild-type mice consistent with previous reports (Figure 5). However, the number of CD8^−^ cDC in *Batf3*^−/−^ varied with 4 of 10 *Batf3*^−/−^ mice showing an increase in CD8^−^ cDC, while another four showed lower numbers of CD8^−^ cDC compared with controls. Variability in CD8^−^ cDC numbers could suggest either multiple interactive effects leading to highly variable numbers, or heterogeneity among the CD8^−^ cDC subset delineated here. The percentage of Ly6C^hi^-inflammatory monocytes also dropped significantly in *Batf3*^−/−^ mice (Figure 4). L-DC, Ly6C^lo^-resident monocytes, and neutrophils displayed no change in percentage due to the *Batf3* mutation. Eosinophils were the only cells that displayed an increase in percentage in *Batf3*^−/−^ mice, but this could be consistent with increased inflammation in these mice.

### L-DC Develop Independently of FLT3L and GM-CSF

Fms-like tyrosine kinase 3 (FLT3) signaling has been described as essential for cDC development in steady-state spleen (54). Administration of FLT3L during *in vitro* and *in vivo* cell development leads to an increase in the number of splenic cDC and pDC (55–57). Similarly, knockdown of *Flt3L* leads to a two- to threefold drop in the number of splenic cDC and pDC in adult mice (54). In order to investigate whether L-DC development is also dependent on FLT3L, the percentage representation of cDC, L-DC and other myeloid subsets was analyzed relative to the total dendritic and myeloid cell population in adult *Flt3L*^−/−^ and wild-type mice.

A sixfold reduction in proportion of CD11c^hi^ cells was observed in *Flt3L*^−/−^ mice compared with wild-type mice, consistent with the literature (58, 59) (data not shown). A significant ~7-fold drop in percentage CD8^−^ cDC among total dendritic and myeloid cells, and a significant drop in percentage of CD8^+^ cDC were observed in *Flt3L*^−/−^ mice compared with wild-type mice (Figure 5A). There was also a significant drop in the percentage of Ly6C^hi^ inflammatory and Ly6C^lo^-resident monocytes in *Flt3L*^−/−^ mice (Figure 5A). In contrast, neither L-DC nor eosinophils were affected by absence of *Flt3L* expression during development. Last, the percentage of neutrophils increased in *Flt3L*^−/−^ mice (Figure 5A). These results predict a role for FLT3L in the development of cDC, and all monocytes, but not L-DC and eosinophils. The proportional increase in neutrophils could reflect inflammation in a mouse model which has reduced immune capacity.

During inflammation, Ly6C^hi^ monocytes can give rise to mo-DC that produce TNF-α and inducible NO synthase. These have been called “Tip-DC” (7, 42, 60). Similarly, granulocyte-macrophage colony-stimulating factor (GM-CSF) can induce formation of inflammatory type mo-DC after addition to cultures of bone marrow progenitors (34, 61, 62). In order to investigate a role for GM-CSF in the development of splenic dendritic and other myeloid subsets, changes in the populational representation of cDC, L-DC and myeloid subsets were investigated in *GM-CSF*^−/−^ mice (Figure 5B). Previous studies have reported no change in the proportion of pDC and cDC in *GM-CSF*^−/−^ mice (63, 64). In this study, however, percentages of both CD8^+^ cDC and CD8^−^ cDC were higher in *GM-CSF*^−/−^ mice (Figure 5B), while percentages of both Ly6C^hi^ and Ly6C^lo^ were significantly lower (Figure 5B). The percentage of eosinophils in *GM-CSF*^−/−^ mice was significantly increased, while the percentage of neutrophils remained unaffected (Figure 5B). Proportional representation of L-DC was not significantly different, although more variable in *GM-CSF*^−/−^ compared with wild type mice.

L-DC development therefore occurs independently of both FLT3L and GM-CSF, while the development of monocyte subsets is clearly dependent on both factors (Figure 5). While cDC subsets are dependent on FLT3L for development, they develop independently of GM-CSF. In fact, in the absence of GM-CSF, there appears to be a compensatory increase in the development of cDC as well as eosinophils (Figure 5). These results distinguish L-DC from both cDC and other myeloid subsets, since L-DC develop independently of BATF3, FLT3L and GM-CSF.

## Discussion

In addressing the issue of myeloid cell subset classification in spleen, a recent paper suggested a unified nomenclature for DC, monocytes, and macrophages based primarily on ontogeny, and secondly by location, function, and phenotype (65). The delineation of splenic Ly6C^lo^ monocytes was made on the basis of the described phenotype and function of previously described resident monocytes in blood (7, 25). Now, it is shown here that the spleen contains distinct populations of Ly6C^hi^CD11c^−^ and Ly6C^lo^CD11c^lo^ monocytes, as well Ly6C^lo^CD11c^−^ eosinophils. The latter population appears to be present in higher number in spleen and so more likely to obscure the Ly6C^lo^CD11c^lo^ monocyte population in the absence of staining for CD11c and the SiglecF marker specific for eosinophils.

In order to better define an *in vivo* equivalent L-DC population in relation to splenic myeloid subsets, it was necessary to analyze dendritic and other myeloid subsets concurrently. Since blood monocytes are the most widely characterized monocytes, their phenotype was used as the starting point for analysis of splenic monocytes (19, 47). Ly6C^lo^ and Ly6C^hi^ monocytes in blood were previously described as CD11b^hi^CD11c^−^MHCII^−^Ly6G^−^ cells (3, 6, 7, 10, 11). Siglec-F, an inhibitory receptor expressed by murine eosinophils (66, 67) proved definitive for analysis of eosinophils in this subset (Figure 2). The CD11b^hi^CD11c^−^MHCII^−^Ly6C^+^Ly6G^−^ subset of cells in spleen was found to comprise a majority (87%) of Siglec-F^+^ eosinophils (Figure 2), with Ly6C^hi^ monocytes clearly Siglec-F^−^ (Figure 2). The results of this study now confirm the definition of splenic Ly6C^lo^ monocytes as CD11c^lo^ cells (9, 10, 68) lying within the Ly6C^+^Ly6G^−^ population.

To further test these initial predictions, the myeloid markers CX_3_CR1 and CD115 were employed. CX_3_CR1 is a known marker of Ly6C^lo^-resident monocytes (7, 25), and CD115 is a marker of bone marrow-derived myeloid cells (43, 44). High levels of CX_3_CR1 were shown to be expressed by CD11c^lo^ cells that were also Ly6C^lo^Ly6G^−^, so confirming their similarity with resident monocytes (Figure 2D). In addition, these cells also expressed CD115 (Figure 2C). Since CD115 was not expressed by the putative L-DC subset, this suggests different lineage origins for L-DC and Ly6C^lo^ monocytes. In line with the literature, Ly6C^hi^ monocytes are CD115^+^, while both neutrophils and eosinophils lacked CD115 expression (Figure 2C).

Phenotypic studies were informative although not definitive in the identification of L-DC in relation to other myeloid subsets. Morphological characterization by May-Grünwald-Giemsa staining was used to confirm predicted subsets. Blood monocytes have a bi-lobate nucleus and minimal cytoplasm devoid of granules (Figure 4) (7, 10). Ly6C^hi^ monocytes in spleen displayed this morphology in line with their blood cell counterparts (Figure 4) (16, 19). Giemsa staining also confirmed that the splenic subset defined here as eosinophils had a characteristic multi-lobate nucleus with orange granules in the cytoplasm, consistent with their phenotypic classification as Siglec-F^+^ eosinophils (Figure 4) (69). The identified Ly6C^lo^ monocyte subset identified as CD11c^lo^ cells displayed morphology consistent with blood monocytes and not DC. These cells had a bi-lobate nucleus with minimal cytoplasm which lacked granules (Figure 3). Therefore morphological studies supported phenotypic studies, showing that spleen resident Ly6C^lo^ monocytes lie within the CD11b^hi^CD11c^lo^Ly6C^+^Ly6G^−^ population. Morphological analysis also showed that all Ly6G^+^ subsets comprised a majority of neutrophils with characteristic multi-lobate nucleus (Figure 4). This was also confirmed by their 7/4 staining (data not shown).

The morphology of splenic DC is known to be to be quite distinct from the first described DC subset of Langerhans cells, which have long membrane projections (70, 71). Here, CD8^+^ cDC were shown to reflect a majority of mononuclear cells with vacuoles evident in the cytoplasm. Similarly, CD8^−^ cDC and up to 60% of the Ly6C^−^Ly6G^−^ L-DC subset also demonstrated similar cDC morphology with cytoplasmic vacuoles (Figure 3). These cells also showed veiled membranes with a few small dendrities, although this cannot be clearly be distinguished on cytospun cells. Combined phenotypic and morphological studies therefore identify L-DC as a subset of CD11b^hi^CD11c^lo^Ly6C^−^Ly6G^−^ cells, with the majority demonstrating the morphology of a DC. A combination of studies shown here, therefore identify L-DC as a distinct cell type, reflecting the *in vivo* equivalent of cells produced in longterm cultures of spleen, LTC-DC. While it has been possible to phenotypically and morphologically identify an *in vivo* counterpart of the *in vitro-*produced L-DC, classification of this cell type as dendritic will be dependent on demonstration of their ability to activate naïve T cells and to cross present antigen as shown previously for *in vitro* produced cells (31, 33).

Knockout mice studies have played a definitive role in identifying genes essential for development. Since none of the mutant mice studied here are embryonically lethal, and none of the genes mutated are crucial for survival, it was possible to obtain essential information about the development of L-DC in relation to other splenic myeloid subsets. CD11b^hi^CD11c^lo^Ly6C^−^Ly6G^−^ L-DC development occurs independently of factors that regulate cDC and monocyte development, including BATF3, FLT3L, and GM-CSF. These findings distinguish L-DC from cDC as a separate lineage of cells, and identify them as distinct from splenic monocyte and granulocyte subsets.

Previously, this lab published evidence that L-DC can arise *in vitro* from self-renewing bone marrow-derived hematopoietic stem cells (HSC) and multipotential progenitors (MPP) following co-culture over splenic stromal lines (72, 73). These findings confirm that L-DC arise from a progenitor distinct from the CDP also present in bone marrow (74). The production of L-DC was unaffected in *Batf-3*^−/−^ mice, suggesting that L-DC develop as a distinct lineage separated from cDC. In *Batf-3*^−/−^ mice, a significant drop in the percentage of inflammatory monocytes was also observed. Recent studies in *Batf-3*^−/−^ mice also showed a defect in the differentiation of CD4 T helper cells into T helper 17 (Th17) cells (75, 76) essential for supporting inflammatory responses involving pathogens or autoimmunity (77, 78). It is interesting to speculate that Ly6C^hi^ monocytes may be important mediators of Th17 cell development from CD4^+^ T helper cells.

The binding of FLT3L to FLT3 triggers the development of cDC from pre-cDC (34, 63, 79), and the knockout of either *Flt3* or *Flt3L* adversely affects cDC development (63, 79). In this study, a significant drop in the production of splenic CD8^+^ cDC and CD8^−^ cDC was observed in *Flt3L*^−/−^ mice (34, 63). FLT3 is also a marker of common lymphoid progenitors (CLP) and CMP (59), such that the delivery of additional FLT3L to mice can lead to an expansion of the monocyte pool along with cDC (58, 59). Expression of the FLT3 receptor on myeloid precursors, and the development of monocytes following FLT3L stimulation suggests that FLT3 receptor tyrosine kinase signaling plays a role in monocyte development. In agreement with this, Waskow et al. (79) showed an increase in the numbers of MDP which give rise to CDP and monocytes following FLT3L stimulation *in vivo* and *in vitro*. A reduction in the number of Ly6C^lo^ resident and Ly6C^hi^-inflammatory monocytes in *Flt3L*^−/−^ mice shown here also supports a role for FLT3 signaling in monocyte development. Since L-DC development is not compromised in *Flt3L*^−/−^ mice, L-DC are distinguishable in terms of development and lineage origin from both cDC subsets, and from the major monocyte subsets in spleen.

Both FLT3L and GM-CSF have been used for *in vitro* expansion of DC. Previously, it was shown that the culture of bone marrow precursors with FLT3L-generated cDC and pDC, while GM-CSF in the same system, generated inflammatory mo-DC (61, 63). *GM-CSF*^−/−^ mice were employed here to determine any role for GM-CSF in the *in vivo* development of L-DC. Deficiency in GM-CSF production did not affect the development of L-DC, suggesting that L-DC are not likely to be an inflammatory type cell dependent on GM-CSF signaling for development from myeloid precursors. By comparison, in *GM-CSF*^−/−^ mice, the percentage of the two main monocyte subsets was reduced, consistent with their development in response to GM-CSF and probably inflammation. The percentage of Ly6C^hi^-inflammatory monocytes was fourfold lower in *GM-CSF*^−^*^*/*^*^−^ mice, while Ly6C^lo^-resident monocytes showed a smaller but significant 1.4-fold reduction. Previous studies in *GM-CSF*^−/−^ mice showed no significant change in peripheral blood myeloid subsets, although alveolar macrophages were found to be defective (80). However, that study did not investigate splenic myeloid subsets to the level of phenotypic detail used here. Under steady-state conditions, the development of cDC occurs independently of GM-CSF (63). In this study, the percentage of CD8^+^ cDC and CD8^−^ cDC was found to be significantly increased in *GM-CSF*^−/−^ mice, although an increase in cDC numbers could occur as a compensatory change in spleen due to reduction in the number of monocytes. This study also showed that eosinophils increased in number in spleens of *GM-CSF*^−/−^ mice, suggesting that the development of eosinophils occurs independently of GM-CSF. An increase in eosinophil number could also reflect an inflammatory or allergic state in *GM-CSF*^−/−^ mice.

In conclusion, a novel dendritic-like cell type termed L-DC has been defined among multiple spleen myeloid subsets as CD11b^hi^CD11c^lo^MHCII^−^CD43^lo^CD115^−^Siglec-F^−^CX_3_CR1^lo^Ly6C^−^Ly6G^−^ cells, resembling *in vitro* grown LTC-DC (32, 33). In defining L-DC, populations of monocytes and granulocytes were also closely considered. Another outcome has therefore been the definition of eosinophils in spleen as CD11b^hi^CD11c^−^Ly6C^+^Ly6G^−^Siglec-F^+^ cells. Ly6C^lo^-resident monocytes in spleen have now been identified more completely as CD11b^hi^CD11c^lo^Ly6C^+^Ly6G^−^CD43^+^CX_3_CR1^hi^CD115^+^Siglec-F^−^ cells. A study of subset prevalence in knockout mouse models confirmed the definition of L-DC in terms of their distinct lineage origin, establishing that L-DC develop independently of *Batf3* expression essential for cDC development, and also independent of the FLT3L/GM-CSF growth factors necessary for cDC and monocyte development in spleen.

## Author Contributions

YH: performance of experiments, analysis and assembly of data, manuscript writing. JT: analysis and interpretation of data, manuscript review. HO: project design and management, planning experiment, analysis and assembly of data, manuscript writing.

## Conflict of Interest Statement

The authors declare that the research was conducted in the absence of any commercial or financial relationships that could be construed as a potential conflict of interest.
