# Supplementary material for: Redefining Myeloid Cell Subsets in Murine Spleen
Source: Front Immunol. 2016 Jan 11;6:652. doi: 10.3389/fimmu.2015.00652 (PMC4707843; doi:10.3389/fimmu.2015.00652)
Supplement: Supplementary file 1 [file Image_1.PDF]

Sup Figure 1

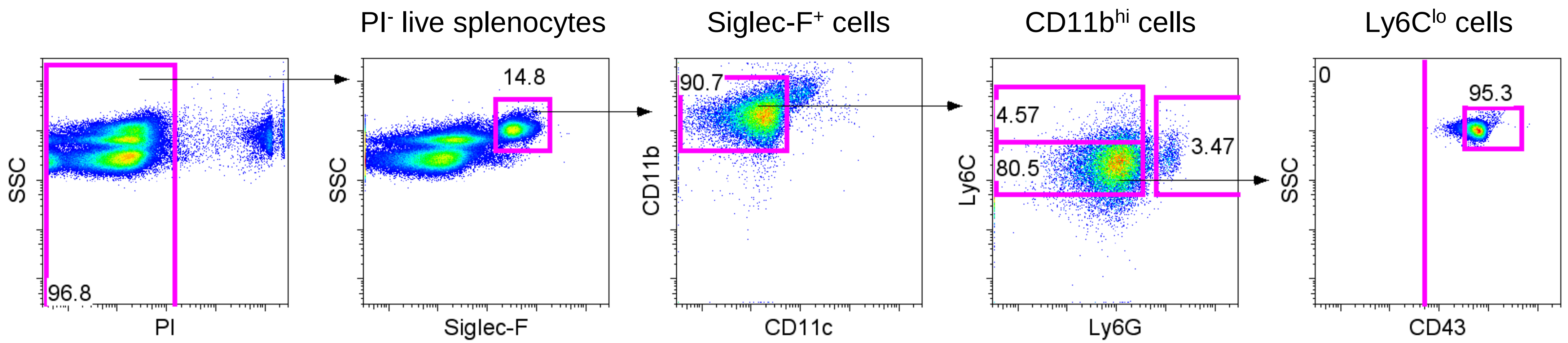

SUPPLEMENTARY FIGURE 1 Backgating of Siglec-F$^+$ cells to identify eosinophil phenotype. Splenocytes were prepared as described in Figure 1 and stained with antibodies to CD11b, CD11c, Ly6C, Ly6G, CD43 and Siglec-F. Prior to flow cytometry, cells were stained with propidium iodide to delineate live (PI$^-$) cells. Siglec-F$^+$ eosinophils were gated and further delineated on the basis of CD11b, CD11c, Ly6C, Ly6G and CD43 expression. The majority of Siglec-F$^+$ cells expressed the phenotype of CD11b^{hi}Ly6C^{lo}CD43^{hi} cells. Gates were set based on fluorescence minus one controls, and numbers in gates represent % specific binding.
